# Facilitators and hinderers for designing augmented reality for ecotourism SME's experiences: A service Design approach

**DOI:** 10.1016/j.heliyon.2024.e24124

**Published:** 2024-01-08

**Authors:** Mario Giraldo, Orietha Rodríguez, Oscar-Naranjo Del Guidice, Mark-Michael Betts

**Affiliations:** a Escuela de Negocios, Universidad del Norte, Barranquilla, Colombia; b Facultad de Ciencias Administrativas y Económicas, Universidad Icesi, Cali, Colombia; c School of Management, Universidad de los Andes, Bogotá, Colombia; d Escuela de Arquitectura, Urbanismo y Diseño, Universidad del Norte, Barranquilla, Colombia

**Keywords:** Service Design, Augmented reality (AR), Tourism, Small and Medium enterprises (SMEs), User experience, User interface, Ecotourism, Latin America

## Abstract

Designing Augmented Reality (AR) throughout service experiences remains under studied in several industries, despite the fact of growing consumer interest and use through different platforms and applications globally. Consequently, there is growing interest in understanding the what, the why and the how for designing AR applications in practice to boost ecotourism experiences, with the purpose of enhancing customer value creation and organizations differentiation. Consequently, the authors conducted an eleven-month practical theoretical study in 10 ecotourism SMEs purposefully recruited in Latin America, adopting service design as a main research framework. Using interviews, contextual analyses, buyer personas, observation, storytelling creation sessions, prototyping sessions and accompaniment sessions as research methods, researchers studied, (1) what were the key facilitators and hinderers for designing AR in practice at the SMEs ecotourism context following a service design lens and, (2) how the inherent principles of service design influence ecotourism SME's for the strategically adoption of AR in their service experiences. The study suggests key elements that can facilitate or hinder designing AR at ecotourism SME's Experiences in practice. Furthermore, the authors suggest a practical protocol for designing AR for ecotourism SMEs from the lens of service design (SD), User Experience (UX), and Augmented Reality (AR). Finally, the study also contributes to shade light on the marketing role and potential adoption of Augmented Reality (AR) in practice in SME's through a service design lens.

## 1 Introduction

Designing and implementing Augmented Reality (AR) developments throughout service experiences remains under studied in several industries, despite the fact of growing consumer interest and use through different platforms and applications globally. Predictions suggest than the business of Augmented Reality (AR), Virtual Reality (VR), and Mixed Reality (MR) development in the service economy is expected to jump for more than 220 billion between 2021 and 2028 worldwide [1]. Consequently, as the use of immersive technologies, including AR, expands through different stages of the service experience, there is growing interest in understanding the what, the why and the how for designing and implementing endearing AR applications in practice to attain an engaging tourism experience throughout its different stages. Several bases have been proposed to identify the gaps, effectiveness, and limitations of AR applications in the tourism experience [[2], [3], [4]], however they are fragmented and inherently confusing in terms of technicalities, methodologies used, targeting, and their effects observed throughout the travel experience. Indeed, exploring, and adapting manifestations for AR design in tourism marketing remains in its infancy and its practical use and application in Small and Medium Enterprises (SMEs) in ecotourism is surprisingly non-existent. Geng and Maimatuerxun [5] suggest innovation in tourism in emerging markets as a key research topic. Also, the importance of the digital economy to achieve sustainability in business, such as tourism, and the revitalization of rural destinations in emerging markets aiming economic and sustainable growth has been currently addressed [6,7]. SMEs also bring with them further particular challenges when encountering new technology implementation, which call for needed research to understand the dynamic nature of its adoption to help them exploit the opportunities offered by those technologies [8]. Furthermore, the application of service design has been acknowledged as a conceptual framework tourism SMEs need to adopt [[9], [10], [11]] to improve the customer experience of their services; consequently, existing service-design methods and tools need to be adapted to the needs and characteristics of tourism SMEs.

There is still limited understanding of the importance, restrictions and interplay of stakeholders’ skills and habits, strategic intent, and service design processes in practice for designing AR initiatives in tourism. In fact, much attention has been paid lately to the use practice theories to underpin tourism studies [[12], [13], [14]], however, they have been understudied when doing AR research in tourism. Ultimately, the purpose of this study is to examine how ecotourism SMEs in the Latin American service sector, particularly hospitality firms, such as hotels and biodiverse attractions, managed the design and implementation of AR initiatives in practice, exploring the needs, habits, actions and requirements associated to those designs, and how those Eco touristic SMEs mitigated occurring resource asymmetries during the different stages of those service design processes. Consequently, the study seeks to address the following research questions: (1) what are the key facilitators and hinderers for designing AR in practice at the SMEs ecotourism context following a service design lens? And (2) how do the inherent principles of service design influence ecotourism SMEs for the strategically adoption of AR in their service experiences?

In addition, we hope to provide insights that may help in the design of tourism services/products and organizational policies for ecotourism SMEs, following the need posed for [12] De Souza Bispo and [13] Bargeman and Richards about a stronger emphasis on understanding tourism practices [13] and the shifting of tourism organizations and individuals to a sort of “practice tourism career” relevant for tourism research in a rapidly changing context. Thus, we also take into consideration the call for understanding habitual ways of dealing with resource asymmetries traditionally posed at the informal and entrepreneurial nature of tourism in developing countries [14] reality present in the ecotourism SMEs context in Latin America.

Five main contributions are presented in this article: first, it provides a comprehensive methodology and research route to study AR design in practice for tourism SMEs through a service design lens. Second, it empirically explores the overlooked practice of designing AR in tourism SMEs, specifically ecotourism, opening a new avenue of research as most studies in AR in tourism focus on the cognitive, emotional, and conditional responses of tourists using AR developments in different types of destinations. Third, the study also empirically addresses the key facilitators and hinderers for designing AR in practice at the SMEs ecotourism. Fourth, it contributes to tourism studies by addressing current real world tourism practices in developing economies that traditionally posed their informal and entrepreneurial nature. Fifth, it introduces a protocol for designing AR for ecotourism SMEs from the lens of service design (SD), User Experience (UX), and Augmented Reality (AR).

We articulate the research article into five sections. First, in the literature review, we start discussing service design as a cross disciplinary research approach that address the complex interplay of stakeholders when applying knowledge and skills at spaces for knowledge co-creation and service development like AR initiatives. We also discuss the adoption of AR research in tourism signaling the gap for AR studies from a more practical and interventionist lens. Second, the methodological route thoroughly discussing research decisions is introduced starting with a general and brief discussion of practice theory as an ontological position. Section three discusses results all over the four phases of the AR design process. The fourth section discusses the facilitators and hinderers for designing AR in ecotourism SME's suggesting a set of countermeasures for addressing the negative impact of the encountered hinderers. Finally, in the conclusion section, implications for theory and practice are presented, introducing a design protocol for AR for tourism SMEs practitioners, as well as specific set of research directions for academics, finishing with the limitations of the study.

## 2 Theoretical framework

### 2.1 Service Design and the tourism consumption Experience

For tourism consumption experiences, hospitality services and sustainable services, service design is a powerful approach that articulates the main body of research in the area [[15], [16], [17], [18]]. Service design as a conceptual framework, has its origins in the service marketing and management literature, and works as an approach which adopts consumer centered innovation, value creation and service development [19,20]. Indeed, service design is about designing the production and consumption experience of services, considering all the interrelated parts of the service ecosystem with an omni-channel approach. It seeks to design service experiences to be feasible, scalable, and financially viable accounting possible pitfalls, miscommunication and misconduct among service users and service providers through a series of practical service encounters [21,22] or touchpoints [23]. Consequently, service design today has become a cross disciplinary research area that involves operations, engineering, design, management, marketing, and sociology.

With the emergence of the Service Dominant Logic SD-L [24,25] and the Service Logic [20,26,27] suggesting that the conventional distinction between goods and services does not matter, as service is considered as the dynamic process of “applying knowledge for the benefit of the parts”, new ways to understand the organization and consumption of services as well as how different service actors access and use their resources [28] creating and co-creating value in value generation process [27,28] through the consumption experience [29,30]. The consumption experience can be seen as an emergent property that results from the interrelationships and overlaps between person, environment, thought, emotion, activity, and value, which can be divided into four phases: previous consumption, purchase, central consumption and remembered consumption or nostalgic [29].

There are different ways to define consumer experiences [31]. Pine and Gilmore [32] argued that consumers seek memorable experiences instead of basic products where they connect with them within four domains of experience determined by (1) the degree of connection of the customer with the environment of the activity and (2) the degree of customer participation, these four areas are entertainment, education, aesthetics, and escapism. Caru and Cova [33] proposed the understanding of experiences based on how realistic or fantastic the environment of the experience is and how active or passive the consumer's performance is in the experience, determining 6 types of performance: (1) performance of skills (e.g., cooking for friends), (2) exciting performances (e.g. surfing), (3) hybrid performance of show and skills (e.g. edutainment) (4) performance of spectacle (e.g. opera, pageantry, concert), (5) hybrid spectacle-festive performance (e.g. car sound competition), and (6) festive performance (e.g. carnival celebrations).

Consequently, it is possible that experiential services, such as tourism and hospitality, might present new challenges for knowledge creation based in how different touchpoints are experienced by service users. Recent work within service management combining sociology, management and marketing has led to understand service encounters as spaces for knowledge creation and service development and co-design through the understanding of them as activity systems [21,22]. Moreover, further developments in areas such as service science [34,35] propose enhancing service design as a research priority, seeing it as an area that aims for cross-disciplinary research [36,37] and transformative intent [38] and innovative service practices [39] seeking sustainable service ecosystems [40].

Additionally, designing services engages researchers into understanding and co-creating challenges and solutions in an exploratory, iterative process and approaching emerging topics abductively. Service Design Thinking allows designing services that meet the needs and exceed the expectations of customers from a stakeholder perspective [41] involving them to create meaningful experiences in the co-design of the service. Its importance lies, according to Polaine et al [42], in the ability of services to adapt to designs that can make the user experience something satisfactory. In the same way, business models oriented to the experience in the services make it possible to evaluate in an efficient way the perception of the user in each one of the stages of the provision of the service and, understand which of them connects little with the users to redesign it and achieve for the system to function optimally.

Finally [41], Stickdorn et al., argue that the fundamental principles of service design are the following: (1) Human-centered, considering the experience of all people affected by the service; (2) Collaborative, integrating different stakeholders in the service design process; (3) Iterative, adopting a methodology that can be constantly adapted during its implementation; (4) Sequential, acknowledging the service must be designed as a sequence of related actions; (5) Real, considering that the needs, the prototype, the user's perception must be realized and considered in reality; and, (6) Holistic, considering the needs of all stakeholders in the system as a whole and not separately.

### 2.2 Augmented reality and its applications in tourism

Enhancing technology mediators (such interactive guides or routes, avatars and mascots, games) as tourism actors is becoming increasingly necessary for service delivery and value creation processes. The development and adoption of such technologies by tourism consumers [[43], [44], [45]], who are being transformed from passive consumers to active co-creators (co-designers) of their tourism experiences [46] is rapidly becoming an essential part of the tourism experience. Applications of AR have increasingly been implemented by the tourism industry, especially for enhancing the experiences for destinations [4,47,48] and museums [49,50]; in promoting education in cultural heritage sector [51,52]; and adopting gamification to engage users at different stages of the touristic experience [53,54]. AR applications account as an angular trend for developing smart tourism experiences to achieve product differentiation and service distinction and distinctiveness over other tourism destinations [55].

The main idea of AR resides on *“augmenting a view of the real world with 2D images or 3D objects”* [50] (p. 230), modifying the user's physical experience by overlapping virtual elements (images, sounds, filters, and virtual objects) beneath their real environment [56,[56], [56], [57], [58]], explains that Augmented Reality (AR) differs from Virtual Reality in how the immersive effect affects users. In Augmented Reality (AR) the immersive effect is partial since the conditioning elements are superimposed on reality, they do not have the same effect as a total virtual reality environment [56,58,59]. Indeed, an important aspect of AR is its ability to *‘enhance a user′s perception of and interaction with the real world’* [56] (p. 3). Finally, AR developments can be used through devices may that be fixed (e.g., interactive displays in museums), mobile (e.g., smart glasses, laptops), or wearable [53,60] and AR solutions success depends on the interactions of users with interfaces and their user experience [58,59].

AR interventions in tourism have proven to be useful in areas related to experience enhancement [61,62,63]; city destination understanding [[64], [65], [63]] travel guidance and city navigation [[66], [67], [68]]; social networking [69]; enhancement of parks and museums experiences [[70], [71], [72]]; and advertising and tourist expectation management [73] and buying behavior [74]. On the other hand [2], acknowledged that AR research in tourism still have gaps and challenges in topics related to usability, lack of AR awareness, time and commitment and unwillingness to accept AR substitutes and Huertas and Iglesia [4] identified lack of knowledge and technical problems as main issues for AR implementation.

Yagol et al. [75], suggest that AR is a developing trend that plays a critical part in transforming traditional tourism. Several studies acknowledge technologies transform tourism management and marketing from a static and utilitarian sense (whereby managers and tourists use technologies as tools) to a transformative conceptualization whereby tourism markets and actors both shape and are shaped by technology [46]. Finally, research about the process and practical implementation of AR initiatives in SMEs tourism sites using participatory and interventionist approaches is lacking [2] and in SMEs in ecotourism is basically non-existent.

## 3 Methods

This research study explores value creation at ecotourism SMEs as sites of practice [21,22,76]. This means that at ecotourism SMEs value is formed in activity systems (subject + cultural and socio-historical context) in which each actor within the activity integrates resources such as physical materials, cultural competences, social communities, motives, power and physical servicescapes through action and its framed by social structures in a wider determined social context [21]. Practice theory is a family of theories with a common focus on practices that facilitate the understanding of social activity [22,76]. Thus, current research in tourism is calling for practice theoretical studies [12,13], to understand the rapidly changing context of tourism [13] and its resource asymmetries [14], As well as, practice theory is considered a hot contemporary marketing topic is the growing role of digital platforms, such as AR, and the resource integration of other technocultural actors [76].

The research study was carried on in Colombia as an initiative of the “4U Alliance”, a university alliance integrated by Universidad del Norte, Universidad EAFIT, Universidad Icesi and CESA, with the purpose of promoting research excellence, university collaboration, university-real sector integration, business development and improvement, and social progress in Colombia. The project started involving a total of ten (10) ecotourism SMEs located in four different geographical regions of the Colombia (i.e., north coast, coffee region) and representing different types of ecotourism initiatives (i.e., eco parks, eco hotels, sports, and biodiverse attractions). These ecotourism SMEs were purposefully recruited [[77], [78], [79]] and selected by the five (5) main researchers of the participant universities, between two (2) and three (3) SMEs by each university, offering them consultancy and follow-up for implementing AR initiatives for their businesses at no economic cost but with research collaboration and involvement. The whole design process, including the development and implementation of AR initiatives, was funded by the 4U alliance. Table 1 shows a brief description of the SMEs studied as indicating which phases of the methodological route was undertaken by them.

**Table 1:** Description and phases of the methodological route undertaken by participant SMEs.

Tourism SMEs	DESCRIPTION	Location	Phases Methodological Route Undertaken			
			E	C	R	I
**Andoke**	Andoke is an educational project around the transformation of the caterpillar into a butterfly. Andoke has a 300 m^2^ Butterfly House covered by polyshade mesh, which houses more than 15 native species of butterflies with all their host plants and other environmental needs. It has a 2000 square meter scale map of Colombia where you can see all the geographic regions, their hydrography, main cities, fauna and flora.	Cali (Valle)				
**AV Café**	Ave Café is a hospitality sme of a coffee grower family from the municipality of Jardín (Antioquia). They offer a coffee tour where they allow you to discover the legacy, the traditions of the collection and production of special coffee, also live the experience of collecting coffee, learn the coffee production process from the seed germination phase to the industrial phase ending the tour with a rest outdoors, enjoying a beautiful view, sunset of the region.	Jardín (Antioquia)				
**Ecoparque Chinauta**	The Chinauta Ecopark is an ecotourism park dedicated to promoting ecological awareness for rest, recreation, learning and fun in Cundinamarca. It has all types of thematic spaces for holding workshops, activities, business, and specialized recreational events. They have a pedagogical outdoor program with educational activities such as managing waste, organic fertilizers use, healthy art, and water as a source of life.	Fusagasuga (Cundinamarca)				
**Reserva Natural El Chochal de Siecha**	Reserva Natural El Chochal de Siecha is a natural reserve whose objective is the preservation of the environment and the species that inhabited it. It is located on the edge of the Guasca Peak, behind a geological gorge crossed by the Siecha River. It is a route of more than 3 ha of protected native forest and more than 5 ha to do rowing and walking activities. There are spaces to interact with animals and cabins and lodges to stay within the reserve.	Guasca (Cundinamarca)				
**Fundación Guanacas**	Fundación Guanacas is an NGO that generates and preserves ecosystems through the conservation and expansion of cloud forests, made up of hectares where various species of wild flora and fauna are found. It has more than 18 water sources, an indigenous pedestrian patio, 133 species of birds and 70 orchids. There are 3 felines of endangered species such as the puma, the oncilla and the ocelot that are essential for the balance of the ecosystem and are threatened by the destruction of their habitat and illegal trafficking.	Santa Rosa de Osos (Antioquia)				
**Fundación Saltamontes**	Fundación Saltamontes is an NGO which objective is to encourage tourism as a powerful tool to reduce poverty, conserve the environment and generate decent jobs. It aims to strengthen the capabilities of rural development ventures in environmental performance, fair trade, compensation programs and the creation of tourist experiences for specialized niches (Scientific Bird Watching, Accessible High Mountain Tours, Heritage Tours).	Salento (Quindío)				
**Niddo Suesca**	Niddo Suesca is a glamping-type hotel located within a nature reserve between the Suesca mountains. There are various species of birds, reptiles, mammals, insects, and species of flora which visitors can interact with, mountains for climbing, and with pedestrian paths for walking to a private rock cliff, the highest point, for contemplating the natural reserve.	Suesca (Cundinamarca)				
**Reserva Natural Bonanza**	Reserva Natural Bonanza is a natural reserve dedicated to the environmental preservation and conservation of the habitat. It offers services such as walks, hikes, camping and eco-experimental workshops which are guided. Within the nature reserve it is possible to interact with natural forests typical of the western mountain range with endemic flora as well as a high diversity of fauna species, also endemic and threatened with extinction.	Jamundí (Valle)				
**Casa Surf**	Casa Surf is an eco-hotel and surf school that aims to integrate people with the preservation of the beaches, understanding nature for nautical activities and promoting surf as an inclusive sport. The eco-hotel and surf school is located in the beaches of Miramar at Puerto Colombia, place that it is, currently, under a strategic transformation as a sport ecofriendly place by the regional government.	Puerto Colombia (Atlántico)				
**Ankua Ecohotel**	Ankua Ecohotel is a boutique hotel which experience seeks to be a solution for families, couples or friends who want to disconnect from urban life, and stay, walk, and learn about the countryside in a tropical dry forest ecosystem. Visitors can stay in ecofriendly hubs, learn agricultural techniques, and taste the gastronomy of the Colombian Caribbean region.	Usiacurí (Atlántico)				

**Fig. 1:**
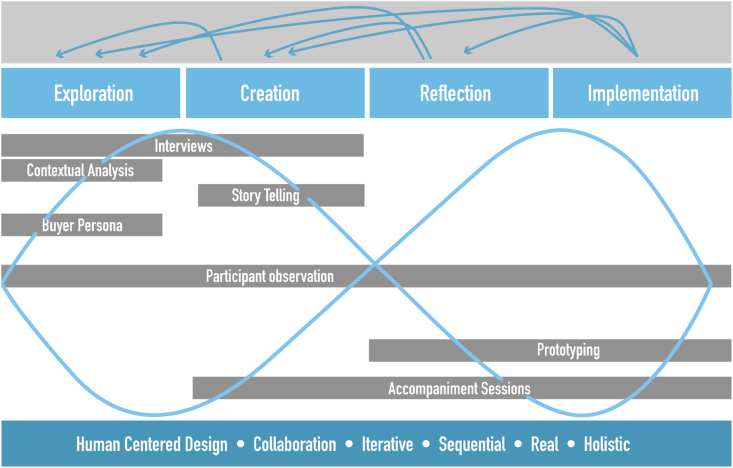
Methodological route research project.

Source: Authors own work

The team of researchers and developers adopted service design as the main research framework to underpin the project as it promotes gaining insight by involving all stakeholders (i.e., companies, consumers, developers, publics) throughout the design process in practice. Indeed, service design enables and democratizes the participation of different stakeholders, helps organizations to see their services from the perspective of consumers and different stakeholders, and recognizes the interests, needs and insights of multiple stakeholders in service ecosystems [41]. Consequently, the methodological route of the research project used the main four service design phases proposed by Ref. [41] Stickdorn et al., joined by the "Double Diamond" model of Design Council (2013) [80] to address divergent and convergent thinking dynamics (Fig. 1). This methodological route took place between February 2022 and December 2022.

To start, in the exploration phase, the design challenge and the expectations of different stakeholders (i.e., research team, SMEs involved, developers) were presented. Aspects related to the culture, structure, strengths, and weaknesses of each of the participating tourism SMEs were identified through interviews, participant observation, contextual analysis, and the creation of buyer personas. Subsequently, in the creation phase, the development of the solution started, by generating and brainstorming ideas from the organizations and the development team, through interviews, participant observation, storytelling sessions and accompaniment sessions. Thirdly, in the reflection phase, the development team presented the prototypes to the organizations and gave instructions for its implementation through accompaniment sessions and participant observation. Finally, in the implementation phase, the ecotourism SMEs implemented the developments and executed communication strategies for the use of the solutions by its clients, supported by accompaniment sessions and participant observation. The data collected in each of the phases were systematized using a matrix titled "matrix for the systematization of information". This tool was designed so that the research team could describe the objectives of each technique used, the roles adopted by the participants, and the factors that facilitated and hindered each of the techniques. In addition, the format allowed the researchers to indicate whether the organization stayed or dropped out of the project. Table 2 shows an example of the use of the matrix for the systematization of information in one of the participant organizations.

**Table 2:** Example matrix of systematization of information.

	Interviews	Storytelling	Buyer Persona	Prototyping	Accompaniment Sessions
Objectives	Present the methodology to be used and the value proposition offered by virtual reality for the fulfillment of the company's objectives.	Choose viable initiatives for the development of augmented reality and the implementation according to their commercial activity and processes.	Design a buyer persona that represents their market segment to design an AR solution that considers their buyer persona perspective.	Represent a digital experience that allows visitors to know the process and the benefits of the brand and the coffee.	Involve the organization in the process of AR design and the storytelling that will engage its visitors.
Roles adopted (Attitudes and willingness to participate)	The project was well received, full support was given and defined dates were established. A presentation and explanation of the process to be followed was made.	DEVELOPER: Rude, uncommunicative. ORGANIZATION: Empathetic, committed.	The organization is very responsive, committed and strives to develop a buyer persona based on evidence and its audiences to achieve the best possible outcome.	The organization received the developments, made good comments and organized them implementation. The result was an accurate digital representation of a coffee bean selection process.	The development of the game has allowed visitors to enjoy and get discounts. It has been very enjoyable when they manage to use the tool.
Facilitators	Enthusiasm and a positive attitude was reflected. They met several times to tell and plan. They identified many fronts from which to develop a story in the case of the company.	The company enthusiastically elaborated the story. The company hired a photographer because the photos they took from the mobile were rejected by the developer.	They welcome all the expectations, since the development of technologies help them to dynamize their business.	The company look for spaces and moments to present to visitors the AR development and encourage them to use it.	The company gave access to their social media to have an accurate measure of the AR design.
Hinderers	The analysis of selection of coffee beans are exhaustive. The company should prioritize to fulfill the project budget and the extension of the project.	At the beginning, they think of the design of the story as a story of the family and them entrepreneurship history.	Lack of knowledge of technical process required to develop the AR properly.	Visitors have problems to use the AR, it is not as intuitive as the developers thought. Users have minimum technological knowledge.	User experience can be improved as they have minimum technological knowledge.
Stay	**X**	**X**	**X**	**X**	**X**
Drop out					

From the ten (10) tourism SMEs that initially started participating in the research project, only five (5) remained for the whole design process and implemented the AR development in their social media spaces. Three (3) organizations dropped out in the exploration phase, two organizations (2) abandoned the project in the creation phase, and five (5) organizations decided not to keep going in the reflection phase. To explore the whys of the SMEs that decided not to continue with the research project, the data obtained in previous phases was analyzed using applied thematic analysis [81]. Applied thematic analysis (ATA) is a rigorous, yet inductive, set of procedures designed to identify and examine themes from data in a transparent and credible form that comprises a bit of grounded theory, interpretivism and phenomenology, synthetized in one methodological framework [81]. ATA is considered a suitable means of analysis when data collection is arranged by multiple researchers and multiple methods.

Answering research questions through ATA requires following the subsequent steps, (1) identification of sources, (2) transcription of data collected, (3) codification, data reduction and linking of data, and (4) ensuring validity and reliability. As for the steps one and two, data was obtained by multiples sources (i.e., researchers, SMEs, developers) and methods (i.e., interviews, contextual analysis, observation notes) that were recorded and transcribed into word files. For codification, data reduction and linking data, the researchers created the "matrix for the systematization of information" (Fig. 1) for initial coding describe the objectives of each technique used, the roles adopted by the participants, and the factors that facilitated and hindered the evolution of the research project. In addition, the format allowed the researchers to indicate whether the organization stayed or dropped out of the project. Initial coding as a category produced empirically is appropriate for virtually all qualitative studies [82].

**Fig. 2:**
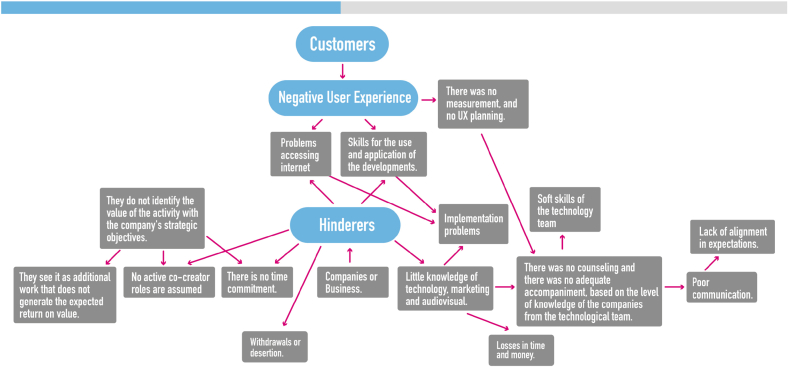
Structural coding research project.

Source: Authors own work.

Subsequently, as a second cycle of coding, the researchers used structural coding [82] using the research questions as the starting point. Structural coding enabled the researchers to compare newly constructed codes during this cycle across cases to assess comparability and transferability [82]. Indeed, as well as initial coding, structural coding was supported through the creation of analytical tree diagrams (see Fig. 2) to identify categories that explicate major patterns and to reveal relationships between categories. Finally, to ensure validity and reliability, intra-coder reliability was adopted, whereby more than one researcher reviewed the themes that emerged [83] and triangulation [84] by data source (i.e., different SMEs), by method (i.e., interviews, field notes, observations, contextual analysis), and by researcher were carried on. The main results of the analysis resulted in the creation of a protocol for the implementation of assertive augmented reality developments for ecotourism SMEs based on the experiences of the project. Fig. 2.

## 4 Results

### 4.1 Exploration

**Fig. 3:**
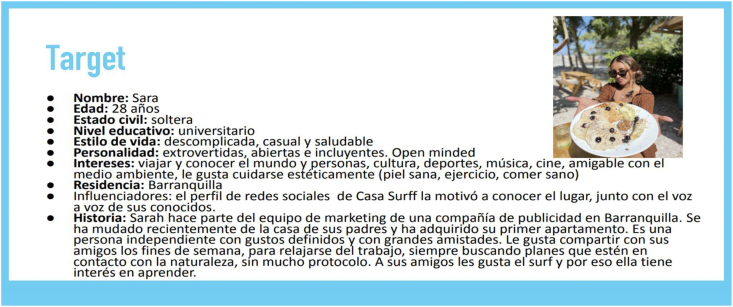
Example of Buyer persona – participant organization.

Source: Authors own work.

**Fig. 4:**
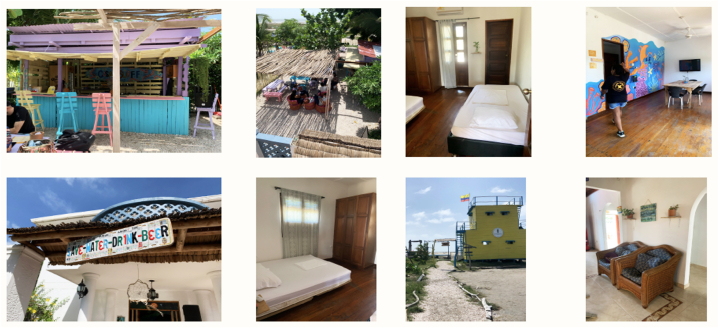
Example of photos of spaces and services - participant organization.

Source: Authors own work.

To begin the exploration phase, an initial interview was held with each of the organizations, during which the design challenge was defined, the expectations of the actors involved were defined and a next meeting was scheduled to conduct a contextual analysis of the organization. The contextual analysis begins with a brief history of the organization and its partners, followed by a review of the company's corporate purpose and size, an analysis of the micro and macro environment, an analysis of the competition and a reformulation of the organization's value proposition, if necessary. Finally, a buyer persona creation session was conducted to characterize the users who will interact with the augmented reality solution (Fig. 3). Throughout this phase, the researchers conducted participant observation in spaces other than those designated for interviews and contextual analysis, taking photographs of the spaces (Fig. 4) and consuming organization's services.

In this phase all the organizations showed great interest, enthusiasm, and positive attitude. However, most of them expressed that they did not have the technical knowledge and needed the support of the team of developers to meet the schedule and stipulated commitments, such as the delivery of photographs and the development of the brief. The organizations that dropped out of this phase did not comply with the contextual analysis session and/or the buyer persona creation, arguing that they did not have the time and staff to properly participate in the project.

### 4.2 Creation

**Fig. 5:**
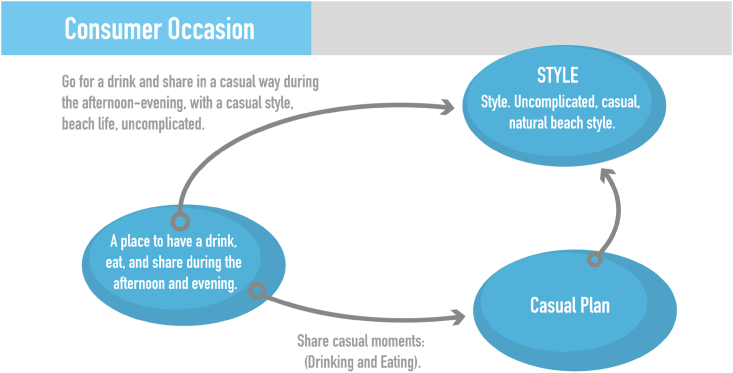
Example of consumption occasion - participant organization.

Source: Authors own work.

The creation phase begun with an interview with each of the organizations in which the information obtained in the exploration phase was examined and it is explained to the participants that they will continue in the next phases. Simultaneously, the researchers continue doing participant observation visits that seek to identify the main consumption occasions for each of SMEs (Fig. 5).

With the information obtained in the exploration phase and the consumer occasion identified through participant observation, The researchers initiated the accompanying sessions for the ideation of possible solutions. The ideas generated were prioritized according to those that can generate greater interest and interaction among consumers and potential customers of the participating companies.

Finally, storytelling sessions are developed in which consumption stories were created with argumentative interconnection, easy to understand, that connect emotionally with consumers and that transmit the value proposition of the organization. The pre-selected ideas and the storytelling were delivered in a brief to the development team, along with photographs and videos of the locations of the participating SMEs for the design of the augmented reality prototype. In this phase, two companies drop out, due to limitations in their technical knowledge, and for having communication and relationship difficulties in with the team of developers. In fact, one of the participating SMEs expressed the following need for patience to the development team:

"I ask you, I don't know how to put it to you, please have patience to understand my position. This topic is new to me. I don't work on it like you do".

This insight helped the research team demonstrating how difficult it was for SMEs to receive support based on their basic level of tech knowledge, bringing communication, emotional and cognitive challenges between the team of developers and the participating organizations.

### 4.3 Reflection

In the reflection phase, the developer team designed the augmented reality solutions and presented them to the tourism SMEs. During the presentation of those solutions, there were identified significant differences between the developer team and the expectations of participating SMEs due to the tourism SMEs' disagreement with the prototype presented. One of the SMEs stated:

"The video of the butterflies' wings is like in the tail, they do not come out well, they do not stay on the back of the users and of the species that were made, the siproeta (A genus butterfly) does not unfold its wings well".

Considering the reflection stage and the iterative nature of the prototyping, follow-up sessions were developed with each of the organizations in which the progress of the prototype and the necessary adjustments to achieve a minimum viable product were reviewed. However, although functional prototypes were achieved, some of the tourism SMEs continued to be dissatisfied with the developers team, expressing that despite having a minimum viable product, factors that were important to them as a company were ignored. One of the participating SMEs expressed:

"Some factors were not taken into account. In the development of the game, the virtual interfaces that arise randomly were not included".

On the other hand, during the validation of the prototypes, difficulties arose due to the difficult access to the internet, which is normal in rural areas in Colombia, and the lack of knowledge of the use of augmented reality solutions by the users of the participating organizations. One of the SMEs stated:

"Users have lack of knowledge to use AR developments, is not easy for them".

Similarly, a different SME stated:

"There are connection problems, many problems with the internet, there was no signal, so they could not access".

**Fig. 6:**
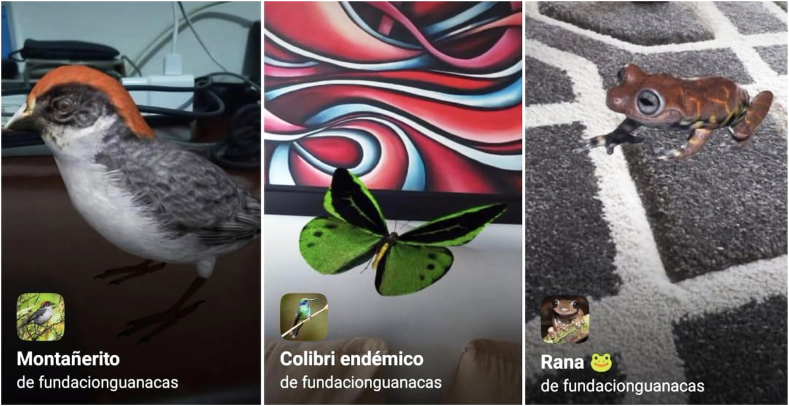
Example of Augmented Reality solution participant organization.

Source: Authors own work.

In this phase, two organizations withdrew from the project due to communication challenges, dissatisfaction with the team of developers, difficulties in accessing the developments and the lack of knowledge of their users about augmented reality. The companies that continued, achieved a minimum viable product for implementation with which they were satisfied and considered that they could generate value to their users (Fig. 6).

The five 5 organizations that continued to the next phase received accompaniment sessions to assist them in the implementation of the augmented reality solutions.

### 4.4 Implementation

In the implementation phase, each of the organizations, with the accompaniment and participant observation of the research team, designed a communication strategy for the use of the solutions by their clients. The communication strategies and the results achieved important milestones in their implementation for 3 months. Table 3 shows the interactions (Open, Capture, Share) achieved by all the organizations throughout the 3 months of implementation. November shows the best results, which is since it was the launching month, and a greater effort was made to communicate the AR solutions. The months of December and January show a decrease with respect to November. However, a stable point of interaction was reached.

**Table 3:** Interactions achieved by all the organizations.

Organization	Interactions	November	December	January
Andoke	Open	1.043	255	283
	Capture	515	102	107
	Share	40	10	11
Avecafe	Open	3.785	641	655
	Capture	206	20	23
	Share	6	1	1
Guanacas	Open	17.500	1.268	1.244
	Capture	2.109	69	77
	Share	408	14	12
Casa Surf	Open	2.874	7.553	5.878
	Capture	184	695	388
	Share	10	40	26
Ecoparque	Open	75.700	1.343	1.539
	Capture	6.107	64	70
	Share	329	3	4

Table 4 shows that the project achieved a total of 100.902 open interactions in November, 11.060 open interactions in December and 9.599 open interactions in January. In relation to capture interactions, the project achieved a total of 9.121 capture interactions in November, 950 capture interactions in December and 665 capture interactions in January. Finally, the project achieved a total of 793 share interactions in November, 68 share interactions in December and 54 share interactions in January (Image 6).

**Table 4:** Total interactions of the project.

Organization	TOTAL		
	Open	Capture	Share
November	100.902	9.121	793
December	11.060	950	68
January	9.599	665	54

Table 5, shows the rate of the interactions of the augmented reality solution per organization. For the period of December all the organizations present a significant decrease in their rate of interactions compared to November. In January, most organizations achieved an increase in their rate of interactions, except for Casa Surf and Guanacas. The interaction rate between November and December is attributed to the communication efforts made to the launch of the AR solutions.

**Table 5:** Rate of interactions of the augmented reality solution.

Organization	Interactions	November–December	December–January
Andoke	Open	−76 %	11 %
	Capture	−80 %	5 %
	Share	−75 %	10 %
Avecafe	Open	−83 %	2 %
	Capture	−90 %	15 %
	Share	−83 %	0 %
Guanacas	Open	−93 %	−2%
	Capture	−97 %	12 %
	Share	−97 %	−14 %
Casa Surf	Open	163 %	−22 %
	Capture	278 %	−44 %
	Share	300 %	−35 %
Ecoparque	Open	−98 %	15 %
	Capture	−99 %	9 %
	Share	−99 %	33 %

On the other hand, the interaction rate between December and January is evidence of the expected use and interaction that the AR solutions will generate within the next months. The negative interaction rate of Guanacas and Casa Surf is caused by the fact that these organizations present greater connectivity problems, and their solutions are among those with the most UX problems.

Despite the high volume of interactions with the AR solutions and the normalization of interactions between December and January, is expected that the interactions decrease if the UX problems are not fixed. Several users have told to the organizations that the AR solutions are not easy to find, are not intuitive, they do not understand how to use it and are difficult to access, due to internet connectivity. One of the SMEs expressed:

"Despite having markers along the route, our consumers do not have access to the tool because there is no internet signal. Moreover, at the points where they can access them, they often do not know how to use or interact with them". (participant organization)

## 5 Discussion

Considering the success of 50 % of the ecotourism SMEs in implementing augmented reality solutions and the desertion of 50 % of the participating SMEs, the following is a description of the facilitators and hinderers elements with the objective of generating a protocol for the implementation of assertive augmented reality developments for ecotourism SMEs in Colombia based on the experiences of the research.

The facilitators elements identified in the organizations that reached the implementation phase are: (1) Business understanding: All parties involved in the research had a clear vision of the business strategy and understood how the implementation of the solution generated value to the stakeholders. (2) Soft skills: The key was the collaborative attitude of the organizations, their ability to maintain assertive communication and build a relationship of understanding with the development team. (3) Co-creation: In the developments in which the organizations and the development team managed to understand their role as co-creators and co-team members without taking a passive position towards the research, the implementation was successful. (4) Involvement of top management: In organizations where, top management was committed to participation, the expected results were achieved and (5) Human talent: Organizations that had fully or partially dedicated collaborators were able to comply with the implementation phase.

The elements that hindered the development of the research and the desertion of 58 % of the organizations are: (1) Technical knowledge: In the organizations that had a low level of digital maturity and low technical knowledge, experience difficulties to understand the information, fulfill the tasks assigned and implement the AR solutions. (2) User experience (UX) design: Failure to consider user experience design, planning and measurement made developments difficult to use, find and access. (3) Alignment of augmented reality development with organizational strategy: Organizations that did not perceive the alignment of the augmented reality solution with their strategic objectives, did not take an active role, did not comply with the proposed schedules, and saw it as additional work. (4) Differential support: Organizations that did not receive support sessions, especially from the technology team, according to their capabilities and resources, dropped out. (5) Connectivity: By not considering the geographic locations, their access to internet and the platforms on which the augmented reality solutions were deployed, there were problems of access and use in several of the organizations (See Table 6).

**Table 6:** Facilitators and hinderers factors for AR implementation in ecotourism SMEs.

Facilitators	Factor	Hinderers
Organizations that have a clear vision of the business strategy and understood how the implementation of the solution will generate value.	**Business Understanding**	Organizations that do not perceive the alignment of the augmented reality solution with their strategic objectives.
Ability to maintain assertive communication and build a relationship of understanding with stakeholders in the project.	**Soft Skills**	Lack of ability to build a relationship of trust, empathy and understanding that facilitate the development of the project. Generating communication and cohesion problems.
Understand their role as co-creators and co-team members, assuming an active, proactive and collaborative role.	**Co-Creation**	Adopt a passive position towards the research, with which they limit themselves to waiting for the work of others and receiving instructions on what to do.
Organizations that have a medium to high level of digital maturity and medium to high technical knowledge, are able to understand the information, fulfill the tasks assigned and implement the AR solutions.	**Tech Knowledge**	Organizations that have a low level of digital maturity and low technical knowledge, experience difficulties to participate properly in the design of AR solutions.
In organizations where top management are committed to participation, the chance of achieve the expected results is higher.	**Top Management Involvement**	If top management do not perceive the project as a priority, even when middle management does. The allocation of resources is not suitable for the success of the project.
When user experience of all stakeholders is of high priority throughout the project, the design of AR solutions, implementation and use, the success rate increases.	**User Experience (UX)**	Failure to consider user experience design, planning and measurement made developments difficult to use, find and access.
Organizations that perceive a close accompaniment according to their needs are more willing to participate in the project and complete it successfully.	**Differential Support**	Organizations that do not receive support sessions according to their capabilities and resources, especially from the technology team, have a higher chance of drop out.
Considering the geographic locations, their access to internet and the platforms on which the augmented reality solutions will be deploy increase the success rate of the project.	**Connectivity**	Not considering the geographic locations, their access to internet and the platforms on which the augmented reality solutions will be deploy, have a lower rate of success.
Organizations that fully or partially assign collaborators to be in charge of the project, are able to comply with the project.	**Human Talent**	Organizations that do not allocate collaborators to be in charge of the project, have difficulties to fulfill the requirements of the project.

### 5.1 Countermeasures

Considering the factors that can hinder the implementation of AR in ecotourism SMEs, we suggest a series of countermeasures that allow organizations to make early decisions to avoid or reduce the negative impact of these hinderers.

1. Ensure the development of a kick-off meeting and regular follow-up meetings with all stakeholders to maintain alignment between the company's strategic goals and the implementation of AR solutions.
2. Validate with a representative of each stakeholder group at each stage of the project that the implementation of the AR solution delivers value to the stakeholders.
3. Create team building workshops to reinforce assertive communication, teamwork, and a collaborative attitude.
4. Identify the soft skills needed to develop the project and conduct a selection process that allows you to have profiles with these skills.
5. As needed, conduct individual, executive, and team coaching sessions to strengthen team members' soft skills, teamwork, conflict resolution, and ensure members' active participation as co-creators.
6. Implement a training program that enables each project participant to have the minimum digital maturity and technical knowledge required for each phase of the project. If necessary, hold extraordinary meetings to ensure that all stakeholders have the necessary competencies and skills.
7. Allocate sufficient space to make, secure, and validate any necessary user experience adjustments at each stage of the project.
8. Identify partners to secure and take corrective action to ensure Internet access and stability of the platform on which the AR solution is/will be deployed.

## 6 Conclusions

### 6.1 Academic implications

The ecotourism SMEs sector is a still understudied case using human centered design. Research approaches for designing AR in tourism have mostly focused on the technical aspects (i.e., issues with terminology, usability, awareness, time commitment) of the developments mostly focusing on measuring and showing results of outcomes using quantitative surveys and experiments [2]. Indeed, following that pattern, recent work quantitatively shows that the development of digital products mediated by service design, in the specific context of the tourism industry [85], yields better results in terms of user experiences when the interface design integrates AR as a semi-immersive resource improving the value proposition of such companies offering tourism services, and additionally establishing a more robust emotional bond between these companies and their users [8,71,[86], [87], [88]].

Following the call about addressing the need for understanding the process for developing AR initiatives in tourism sites using participatory and interventionist approaches [2,[12], [13], [14],^46^], our study sheds light about the actual practice for doing a whole design cycle for creating such developments in tourism SMEs through a service design lens. Results suggest multi stakeholder design barriers that hinder developing AR solutions for ecotourism SMEs which are suggested to be addressed with a practical protocol (see practitioner's implications) for generating more sustainable service offers in this business sector. Moreover, it is important to rely on the iterative capability of service design that enables companies and customer to continually improve AR solutions that meet the expectations of all stakeholders and adapt to volatile changes in technology and society. However, further research is still needed for addressing design needs and practices of each one of these stakeholders at different levels. For example, the impact (i.e., emotional, educational, economic, social, transformative) implementing AR initiatives in ecotourism SMEs without considering how stakeholders will adapt to these redesigned offerings.

Moreover, the research enhances comprehension and addresses deficiencies related to the integration of augmented reality (AR) in the tourism industry [2] in practice. The study indicates that technological developments alone are not sufficient to enable consumers to play an active role as co-creators (co-designers) to enhance their tourism experience through AR [[43], [44], [45]] in practice. It is required to meticulously design each touchpoint of the experience with the fundamental principles of service design [41] to ensure that the knowledge and technical capabilities [4] of all stakeholders and their experiences are taken into account to ensure an appropriate user perception and interaction [56] that enhances their tourism experience.

For such reason, this article introduces a methodological route which advances the conceptualization of designing AR for ecotourism by bridging multidisciplinary perspectives of UX, design thinking and service design. To this end, the methodology proposed offers a process, iterative and practical model for identifying barriers at the different stages of the design process, allowing the reflective development and adjustment of new AR products in ecotourism SMEs from user and service experience perspectives.

### 6.2 Practitioner's implications

The methodological route use in this study also includes a reflection and visualization highlighting the different facilitator and hinderer factors thet affect AR development throughout the service design creation at ecotourism SMEs. The methodological route for developing AR interventions in ecotourism SMEs, proposed above, require the active and open involvement of designers, users, technical profiles and suppliers as well as business owners, without mentioning governmental agencies, throughout the entire AR service creation process.

Consequently, based on the facilitators and hinderers identified and visualized throughout this research study, we suggest the following protocol for the implementation of sustainable AR developments for ecotourism SMEs from the lens of service design.

1. Business understanding and Strategy: All parties involved understand the project, have a clear vision of the business strategy and understand how the implementation of the solution generates value to the stakeholders.
2. Soft skills: The parties involved must ensure a collaborative attitude, assertive communication and team cohesion, and it is important to have a product manager who oversees the interaction between the parties and the development of the project according to the expectations of the parties.
3. Co-creation: Participants should take an active role as co-creators of the solution throughout the process.
4. Technical knowledge: An assessment of the organizations intending to participate should be performed in order to ensure that they have the minimum digital maturity and technical knowledge for the project. If they do not have the minimum level of digital maturity and technical knowledge. A previous awareness and training phase should be included in the project in order to reach the minimum level for their active and appropriate participation in the project.
5. Top management involvement: Participating organizations should sign collaboration agreements with senior management to ensure commitment to the schedule, resources, deliverables and responsibilities in general.
6. Human talent: Organizations must assign a full or part-time collaborator to be responsible for the success of the project.
7. UX user experience design: The project must consider the design, planning and measurement of the user experience in the creation, reflection and implementation phase.
8. Differential support: The organizations require that the support sessions, especially those of the technology team, be designed with a differential approach in which each of the organizations will receive support according to their capabilities and resources.
9. Connectivity: It is important to consider the geographic locations and their connectivity in the development of augmented reality solutions and the platforms on which they will be deployed to ensure their availability.

This research contribution relies on, first (1), identifying the key facilitators and hinderers for implementing AR in the SMEs ecotourism following a service design lens in through a real implementation in practice using ethnographic methods; second (2), proposing a protocol using the inherent principles of Service design [41], design thinking [89], User Interface (UI), User Experience (UX), Usability and Augmented Reality [58,66,[90], [91], [92]] for the strategically adoption of AR in ecotourism SMEs service experiences; third, (3) the study also contributes to the understudied marketing role and potential for designing and implementing Augmented Reality (AR) developments throughout service design lens in SMEs and; (4) finally, results indicate that designing ecotourism services based on AR can enhance customer satisfaction [4,12,41,47], trust [93], and differentiation [55].

### 6.3 Future research directions

Finally, the limitations of this study are that it was conducted only in Colombia and that the implementation period was only three months and took into consideration a single loop of iterations. Although the chosen methodology allows an in-depth analysis, it could be strengthened by a future quantitative approach. In addition, for future studies, it is suggested to use the protocol to validate its relevance, to apply the protocol in augmented reality projects in other industries, to expand the number of loops for iterations for designing developments, and to generate a quantitative instrument that allows the measurement of each of the elements to project the success rate of the development. Furthermore, to enhance comprehension of the key factors that facilitate or hinder AR design in practice, the inherent principles of service design that influence the strategic adoption of AR in service experiences, and to address the existing gaps. We suggest potential research questions.

1. Which are the touchpoint stimuli that motivate tourism consumers to become active co-creators (co-designers)?
2. How can the usability of AR experiences be improved in ecotourism environments with limited internet access?
3. What are the most relevant competitive attributes in the design of smart tourism experiences based on AR?
4. What ethical dilemmas arise when modifying users' perception of and interaction with the real world?

## CRediT authorship contribution statement

**Mario Giraldo:** Writing – review & editing, Writing – original draft, Project administration, Funding acquisition. **Orietha Rodríguez:** Supervision, Project administration, Investigation, Funding acquisition, Data curation, Conceptualization. **Oscar-Naranjo Del Guidice:** Writing – review & editing, Writing – original draft, Methodology, Investigation, Formal analysis, Data curation, Conceptualization. **Mark-Michael Betts:** Writing – review & editing, Writing – original draft, Methodology, Conceptualization.

## Declaration of competing interest

The authors declare the following financial interests/personal relationships which may be considered as potential competing interests: Mario Giraldo reports financial support was provided by Alianza 4U (10.13039/501100004245Universidad del Norte, 10.13039/501100002411Universidad Icesi, 10.13039/100013404Universidad EAFIT, CESA). Orietha Rodriguez reports financial support was provided by Alianza 4U (10.13039/501100004245Universidad del Norte, 10.13039/501100002411Universidad Icesi, 10.13039/100013404Universidad EAFIT, CESA). If there are other authors, they declare that they have no known competing financial interests or personal relationships that could have appeared to influence the work reported in this paper.
